# Effect of Yogurt Ice Cream on the Viability and Antidiabetic Potential of the Probiotics *Lactobacillus acidophilus*, *Lacticaseibacillus rhamnosus*, and *Bifidobacterium animalis* subsp. *lactis* after In Vitro Digestion

**DOI:** 10.3390/foods12234373

**Published:** 2023-12-04

**Authors:** Rinrada Talearngkul, Sudathip Sae-tan, Jintana Sirivarasai

**Affiliations:** 1 Master of Science Program in Nutrition, Faculty of Medicine Ramathibodi Hospital and Institute of Nutrition, Mahidol University, Bangkok 10400, Thailand; rinrada.t16@gmail.com; 2 Department of Food Science and Technology, Faculty of Agro-Industry, Kasetsart University, Bangkok 10900, Thailand; fagists@ku.ac.th; 3 Nutrition Division, Faculty of Medicine Ramathibodi Hospital, Mahidol University, Bangkok 10400, Thailand

**Keywords:** probiotic, in vitro digestion model, dairy food, α-amylase, α-glucosidase

## Abstract

Probiotics can ameliorate type 2 diabetes mellitus (T2DM) via several mechanisms such as by decreasing inflammatory cytokines and increasing pancreatic β-cell functions. Another targeted mechanism for managing T2DM involves inhibiting α-amylase and α-glucosidase, which exhibit antioxidant activity and affect carbohydrate metabolism by delaying carbohydrate digestion, thus mitigating glucose in the circulation. Dairy products are effective matrices for delivering probiotics through the gastrointestinal tract. We compared the viability and antioxidant activity of the probiotics *Lactobacillus acidophilus LA-5*, *Lacticaseibacillus rhamnosus GG*, and *Bifidobacterium animalis* subsp. *lactis* in yogurt ice cream after in vitro digestion and compared α-amylase and α-glucosidase inhibition activities. *Lacticaseibacillus rhamnosus GG* had the highest viability after in vitro digestion (oral, gastric, and intestinal). *Lactobacillus acidophilus LA-5* and *Lacticaseibacillus rhamnosus GG* exhibited the highest percentages of α-glucosidase (16.37% ± 0.32%) and α-amylase (41.37% ± 0.61%) inhibition. *Bifidobacterium animalis* subsp. *lactis BB-12* and *Lactobacillus acidophilus LA-5* showed the highest antioxidant activities via the α,α-diphenyl-β-picrylhydrazyl free radical-scavenging method and ferric-reducing antioxidant power assay, respectively. These findings suggest that yogurt ice cream can provide a suitable matrix for the delivery of probiotics from dairy culture to promote intestinal homeostasis with probiotic benefits in the host as well as a potential functional food to help reduce postprandial hyperglycaemia.

## 1. Introduction

Type 2 diabetes mellitus (T2DM) is among the most common non-communicable diseases and a serious public health concern. A current report from the International Diabetes Federation indicated that the global prevalence of T2DM in adults was 10.5% (536.6 million people) in 2021 and that 783.2 million people (12.2%) worldwide would be living with diabetes by 2045 [1]. Furthermore, between 2021 and 2045, middle-income countries are expected to have the highest relative increase in diabetes prevalence (21.1%), followed by high-income (12.2%) and low-income (11.9%) countries [1]. T2DM is characterized by chronically elevated blood glucose (hyperglycemia) and elevated blood insulin (hyperinsulinemia) owing to defective insulin secretion by pancreatic β-cells and the inability of insulin-sensitive tissues to respond appropriately to insulin [2]. Lifestyle, nutritional factors, physical activity, and gut dysbiosis are significant factors in pathological T2DM [2].

Gut dysbiosis is primarily associated with compositional and functional alterations of the gut microbiome, resulting in decreased microbiota diversity, loss of beneficial microorganisms, or an overgrowth of harmful microorganisms [2]. A potential approach to restoring the microflora balance is the use of probiotics. Probiotics have been shown to enhance glycemic control by increasing the production of short-chain fatty acids (SCFAs) such as acetate, propionate, and butyrate [3]. Increasing these SCFA levels promotes essential β-cell-protective effects, helps maintain glucose homeostasis, improves insulin sensitivity, and reduces proinflammatory cytokines and oxidative stress [3].

The World Health Organization defines probiotics as “live microorganisms which, when administered in adequate amounts, confer a health benefit on the host”. At the time of consumption, the minimum amount of probiotic bacteria required to confer a health benefit is 106 viable cells/g/mL of product [4]. Tonucci et al. (2015) discovered that patients with T2DM who consumed probiotic-fermented milk (containing 10^9^ colony-forming units [CFU] of *Lactobacillus acidophilus LA-5* and 10^9^ CFU of *Bifidobacterium animalis* subsp. *lactis BB-12* had lower HbA1C [5]. A previous study assessed the changes in mice receiving daily *Lacticaseibacillus rhamnosus GG (LGG)* treatment at 1 × 10^8^ CFU/mouse for 4 weeks. The results showed that *LGG* treatment improved insulin sensitivity by lowering endoplasmic reticulum and oxidative stress [6]. Additionally, LGG exhibits α-glucosidase inhibitory activity in vitro [7]. Controlling postprandial glucose by reducing carbohydrate digestibility by controlling the activity of the hydrolyzing enzymes amylase and glucosidase is considered an effective treatment for T2DM. Because both enzymes participate in carbohydrate digestion, inhibiting them may delay the breakdown of carbohydrates, thus preventing glucose from entering the circulation and functioning as a viable prophylactic treatment for hyperglycemia complications [8]. Probiotic strains produce proteolytic enzymes, resulting in increased bioactive peptides that can block these enzymes and decrease postprandial blood glucose levels with possible positive effects on hyperglycemia and T2DM [9,10]. Mushtaq et al. (2019) showed that kalari cheese with added probiotics had better α-glucosidase and α-amylase inhibition activity than did cheese without added probiotics at all concentrations [10].

In association with chronic hyperglycemia, the T2DM pathogenesis involves increased formation of free radicals and a lack of antioxidant protection [11]. Oxidative stress is increased in patients with diabetes owing to various molecular mechanisms, including glucose autoxidation, impaired antioxidant levels and enzyme activities, and interactions of advanced glycation end products with specific cellular receptors, thus leading to insulin resistance, inflammation and diabetic vascular complications [12]. Probiotic-fermented milk supplemented with *Lactobacillus acidophilus, LGG* and *Bifidobacterium animalis* subsp. *lactis* has shown antioxidant properties with significant probiotic viability (>7 log10 CFU/g) and high antioxidant capacities [13].

Ice cream is the most popular frozen dairy product and can be developed as a functional food with probiotics. Yogurt ice cream is a healthy food with nutritional value owing to its protein, fat, lactose, and mineral contents. Yogurt consumption has been inversely associated with the risk of T2DM [14]. The composition of ice cream, including milk protein, fat, lactose, and other compounds, makes it a suitable substrate for probiotic microorganisms in the gastrointestinal tract [4].

In this study, we used in vitro digestion to evaluate the effect of yogurt ice cream on the viability and antidiabetic properties of *Lactobacillus acidophilus*, *Lacticaseibacillus rhamnosus*, and *Bifidobacterium animalis* subsp. *lactis*. Our hypothesis is that yogurt ice cream as a delivery vehicle is associated with the number of viable probiotics after stages of in vitro gastro-intestinal digestion, and that different probiotics in yogurt ice cream have different antidiabetic properties.

## 2. Materials and Methods

### 2.1. Materials

Yogurt ice cream was prepared using: sugar (sucrose, Lin, Bangkok Thailand), partly skimmed milk powder (PMP), maltodextrin and stabilizers and emulsifiers (all supplied by Miss Ice Cream, Bangkok, Thailand), salt (Prung Thip, Surat Thani, Thailand), citric acid (Miss Ice Cream, Bangkok, Thailand), whole milk (Meiji, Bangkok, Thailand); whip cream (RICH’S, Bangkok, Thailand); and yogurt (Yolida, Nakhon Ratchasima, Bangkok, Thailand). The commercial probiotic cultures used in the study were *Bifidobacterium animalis* subsp. *lactis* BB-12, *Lacticaseibacillus rhamnosus* and *Lactobacillus acidophilus* LA-5 (Chr. Hansen, Honsholm, Denmark). One gram (1.0 g) of a fine, standardized powdered, freeze-dried culture contained 1 × 10^11^ CFU of *Lactobacillus acidophilus LA-5*; 1 × 10^11^ CFU of *Bifidobacterium animalis* subsp. *lactis* BB-12; and 5 × 10^11^ CFU of *Lacticaseibacillus rhamnosus*.

In vitro digestion was performed using KH_2_PO_4_ (Vivantis Inc., Oceanside, CA, USA), KCl (Vivantis Inc.), NaHCO_3_ (European Distribution Center, Erembodegem-Aalst, Belgium), NaCl (Merck, Darmstadt, Germany), MgCl (Univar, Auckland, New Zeland), (NH_4_)_2_CO_3_ (KemAus, Cherrybrook, Australia), CaCl_2_·2H_2_O (KemAus), pepsin from porcine gastric mucosa, α-amylase and pancreatin from porcine pancreases, and porcine bile (Sigma-Aldrich, B8631). MRS broth (Difco, Sparks, MD, USA) and agar (Scharlau, Arcadia, WI, USA) were used for total bacterial counts.

HCl, sodium phosphate buffer, and NaCl (Merck; Darmstadt, Germany); α-amylase and α-glucosidase (Sigma-Aldrich; St. Louis, MO, USA); and starch solution, dinitro salicylic acid, tartrate solution, and p-nitrophenyl-α-D-glucopyranoside solution (pNPG) (Sigma-Aldrich; St. Louis, MO, USA) were used for the α-amylase and α-glucosidase inhibition assay. 1,1-diphenyl-2-picrylhydrazyl radical (DPPH), 2,4,6-Tris(2-pyridyl)-5-triazine (Sigma-Aldrich; St. Louis, MO, USA), acetate buffer, ferric chloride hexahydrate, HCl, and ethanol were used for the DPPH-radical scavenging and ferric-reducing antioxidant power (FRAP) assays.

### 2.2. Yogurt Ice Cream Preparation

A mixture of 132 g milk and 125 g whip cream was placed in a stainless steel pot and heated to 55 °C. Next, the mixture of solid ingredients (100 g sugar, 42 g PMP, 35 g maltodextrin, 4.5 g SEP, 0.2 g salt, 4 g citric acid) and 580 g yogurt was added and blended until the mixture became clear. This yogurt ice cream base mix was agitated in a blender for 2 min, then further heated to 80 °C (pasteurized) for 2 min. Each probiotic strain was added to the mixture and then cooled to 41 °C to 43 °C, and then further incubated at 4 °C for 24 h. The now-cooled and fermented mix was finally frozen in an ice cream machine for 12 min per batch. The percent overrun of this original yogurt ice cream was 7.4 ± 2.3%.

### 2.3. Nutritional Information for the Yogurt Ice Cream

Nutritional values for energy, total fat, protein, carbohydrate, sugar, sodium, ash, and moisture in the yogurt ice cream were analyzed by the Asia Medical and Agricultural Laboratory and Research Center. The analytical methods followed the Association of Official Agricultural Chemists (AOAC) (1993), AOAC (2012), and AOAC (2019) guidelines and methods of analysis for nutrition labeling.

### 2.4. In Vitro Digestion

The simulated digestion was performed as previously described [15,16]. Yogurt ice cream with probiotics (2.5 g) was randomly sampled from three cups to determine the viability of the probiotics before and during in vitro digestion. This method comprised consecutive phases resembling various conditions through the gastrointestinal tract (oral, gastric and intestinal digestion). For oral digestion, 2 mL of stock simulated salivary fluid (SSF) solution, 1.5 mL of α-amylase solution (75 U/mL), 12 µL of 0.3 M CaCl_2_ and 488 µL of water were added to each sample. The mixture of dissolved ice cream and saliva was incubated in a shaking water bath at 37 °C for 2 min. For the gastric phase, the process was initiated by adding 3 μL of CaCl_2_ and 347 μL of water into the oral bolus. The mixture was adjusted to pH 3 with HCl (6 M), and 4.55 mL of pepsin solution was added to the simulated gastric fluid (SGF). The mixture was incubated in a shaking water bath at 37 °C for 2 h (pH adjusted every 30 min to maintain pH 3). The intestinal phase was initiated by adding 20 μL of CaCl_2_ and 655 μL of water into the gastric chyme. The mixture was adjusted to pH 7 with NaOH (1M), and then 8 mL of pancreatin solution and 1.25 mL of bile extract were added to the simulated intestinal fluid (SIF). The mixture was incubated in a shaking water bath at 37 °C for 2 h (pH adjusted every 30 min to maintain pH 7). At the end of each phase, the sample was collected and put in an ice bath to stop the enzymatic reaction.

### 2.5. Preparation of Yogurt Ice Cream Probiotic Water Extracts (YIEs)

YIEs were prepared as per Shori et al. [16]. Briefly, 10 g of yogurt-probiotic ice cream was homogenized with 2.5 mL sterile distilled water. Then, the targeted homogenates were acidified to pH 4 with HCl (0.1M), heated in a water bath at 45 °C for 10 min, and centrifuged (5000× *g*, 10 min, 4 °C). The supernatant was then adjusted to pH 7 with NaOH (0.1 M) and centrifuged (5000× *g*, 10 min 4 °C) to precipitate the proteins and salts. Finally, the supernatants were collected, refrigerated at 4 °C, and used within 12 h of preparation.

### 2.6. Microbiological Analysis

To determine the viability of *Lactobacillus acidophilus* LA-5, *Bifidobacterium animalis* subsp. *lactis* BB-12, and LGG in yogurt ice cream before and during in vitro digestion, the ice cream was analyzed by the Thailand Institute of Scientific and Technological Research at the Biodiversity Research Center. The details and methods followed ISO 29981/IDF 220: 2010, Milk products–Enumeration of presumptive bifidobacteria and lactobacillus–Colony count technique and ISO 29981/IDF 220: 2010 and ISO 7218 Microbiology of food and animal feeding stuffs–General requirements and guidance for microbiological examinations.

### 2.7. α-Amylase Inhibition Assay

The α-amylase inhibition assay was performed as previously described [17]. First, 125 µL of YIEs and 125 µL of α-amylase (2 units/mL) were mixed and incubated at 37 °C for 5 min. Next, 250 µL of 0.2% (*w*/*v*) of starch was added and dissolved in the deionized water, and the reaction mixture was further incubated at 37 °C for 5 min. The reaction was stopped with 250 µL of dinitrosalicylic acid color reagent. The mixture was incubated in a boiling water bath for 5 min and cooled to room temperature. To determine the α-amylase inhibition activity, the absorbance of the mixture at 540 nm was measured with a microplate reader (TECAN, Männedorf, Switzerland). Yogurt ice cream without probiotics was used as a control. The same process using deionized water instead of enzyme solution was followed to generate an analytical blank. Acarbose (Sigma-Aldrich, St. Louis, MO, USA) was used as a positive control. The enzyme inhibition percentage was calculated as follows:

Inhibition % = [(absorbance of control − absorbance of extracts)/absorbance of control] × 100.

### 2.8. α-Glucosidase Inhibition Assay

The α-glucosidase inhibition assay was performed as per Orathai et al. [17]. Briefly, 20 µL of YIEs, 20 µL of α-glucosidase (1 unit/mL), and 460 µL of 0.1 M potassium phosphate buffer solution (pH 6.8) were incubated in a water bath at 37 °C for 20 min. Next, 200 µL of 1 mM pNPG was added to initiate the reaction and further incubated at 37 °C for 15 min. Next, 500 µL of 1M Na_2_CO_3_ in deionized water was added. To determine the α-glucosidase inhibition activity, the absorbance of the mixture at 405 nm was measured with a microplate reader (TECAN). Yogurt ice cream for the control and blank were prepared as per Section 2.7, with acarbose as the positive control. The enzyme inhibition percentage was calculated as per the above equation.

### 2.9. Antioxidant Activity by DPPH Inhibition Assay

DPPH inhibition was determined as previously described [18]. Briefly, 250 µL of YIEs was mixed with 3 mL of DPPH (60 mmol/L in ethanol). The reaction mixture was vortexed thoroughly and allowed to stand at room temperature. The absorbance of the mixture was measured spectrophotometrically at 517 nm. Yogurt ice cream without probiotics was used as a negative control; Trolox was used as a positive control.

### 2.10. FRAP Assay

The iron ion reduction power was evaluated as per Vicenssuto et al. [19]. First, FRAP reagent solution was prepared with 10 mL of acetate buffer (300 mM) and adjusted to pH 3.6 by adding acetic acid. Next, 1 mL of ferric chloride hexahydrate (20 mM) dissolved in distilled water and 1 mL of 2,4,6-Tris(2-pyridyl)-s-triazine (10 mM) dissolved in HCl (40 mM) were mixed in. YIEs (50 µL) was added to 350 µL of FRAP reagent solution. The reaction mixtures were stirred and then incubated at 37 °C for 20 min. Yogurt ice cream without probiotics was used as a negative control; Trolox was used as a positive control. The absorbance was measured with a microplate reader (TECAN) at 595 nm.

### 2.11. Statistical Analysis

Data analysis was performed using SPSS for Windows, version 23. There were three replicates for each analytical measurement. Each experiment was repeated three times for calculation of the mean and standard deviation for each sample. One-way analysis of variance was used to compare means between groups; Duncan’s test was used for pairwise comparisons. Differences were considered significant at *p* < 0.05.

## 3. Results and Discussion

### 3.1. Nutritional Information for the Yogurt Ice Cream

Functional ice cream with nutrients and health benefits is a growing trend in dairy products. Yogurt ice cream (80 g) provides 140 kcal of energy, 4.0 g protein, 5.0 g total fat, 10.0 mg cholesterol, 19 g total carbohydrates, 15 g sugar, and 60 mg sodium. Those quantities constitute 7% of the energy, 23% of the sugar, 8% of the fat and 3% of the sodium recommended as Thai Recommended Daily Intakes for people over 6 years of age resistant on a 2000 kcal diet.

Previous studies proposed that the underlying mechanisms related to dairy consumption for the decreased risk of T2DM involve amino acids and bioactive peptides derived from milk proteins. These nutrients can regulate postprandial glucose levels by delaying gastric emptying and by stimulating insulin secretion from β-cells [20]. Because fermented dairy products such as yogurt contain probiotic bacteria, they may provide additional health benefits. We evaluated the antidiabetic effects of yogurt ice cream. A previous meta-analysis found that dairy foods, particularly ice cream (relative risk at 10 g/d: 0.81; 95% confidence interval [CI]: 0.78–0.85; *p* < 0.001) and yogurt (relative risk at 80 g/d: 0.86 compared with 0 g/d; 95% CI: 0.83–0.90; *p* < 0.001), may help prevent T2DM [21].

### 3.2. Viability of Probiotics in Yogurt Ice Cream before and after In Vitro Digestion

Inoculating 108 CFU/g probiotics into mixed ice cream provided yogurt ice cream with predigestion viable counts of ≥ 6 log CFU/g of *Lactobacillus acidophilus* LA-5, *Bifidobacterium animalis* subsp. *lactis* BB-12, and LGG, indicating that the product was a probiotic. Table 1 shows the number of bacterial cells in yogurt ice cream with each probiotic before and after exposure to each step of in vitro digestion. At the end of the oral phase, the probiotic viability decreased significantly in all three groups compared with those of the predigestion phase (yogurt ice cream *Lactobacillus acidophilus* LA-5 [YI-LA-5]: 6.61 ± 0.02 vs. 5.12 ± 0.02 log CFU/g, yogurt ice cream *Bifidobacterium animalis* subsp. *lactis* BB-12 [YI-BB-12]: 6.23 ± 0.03 vs. 4.53 ± 0.57 log CFU/g, and yogurt ice cream LGG [YI-LGG]: 7.68 ± 0.04 vs. 6.35 ± 0.09 log CFU/g, all *p* < 0.05). Several factors in yogurt ice cream production, including the freezing process, thermal shock, osmotic pressure within the cells, incorporation of oxygen into the mixed ice cream, and mechanical action during air mixing, can reduce the probiotic viability [4]. YI-BB-12 had the lowest cell count as determined by probiotic viability during production and storage before in vitro digestion. This may be because *Lactobacillus* strains are more resistant to hazardous conditions during the ice cream manufacturing process than are bifidobacterial strains [4].

After the gastric phase, the viability of *Lactobacillus acidophilus* LA-5 and *Bifidobacterium animalis* subsp. *lactis* BB-12 decreased by approximately 1 log, and viable counts were <10^6^ CFU/g. However, the differences were significant only in *Bifidobacterium animalis* subsp. *lactis* BB-12 (4.53 ± 0.57 vs. 3.11 ± 0.12 CFU/g, *p* < 0.05). Conversely, LGG did not decrease significantly compared with that in the oral phase (6.35 ± 0.09 vs. 6.23 ± 0.08 log CFU/g). For the duodenal phase, YI-BB-12 and YI-LGG exhibited the highest and lowest sensitivity, respectively, to simulated intestinal stress (Table 1). Viable cell counts did not decrease significantly for *Lactobacillus acidophilus* LA-5 or LGG throughout digestion. After simulated passage through the gastrointestinal tract, LGG yielded 6.04 log CFU/g, an amount considered adequate for intestinal colonization. Conversely, the intestinal viability of *Lactobacillus acidophilus* LA-5 was 4.11 log CFU/g, which is considered low and likely insufficient to provide probiotic benefits. For *Bifidobacterium animalis* subsp. *lactis* BB-12, the initial viability was 6.61 log CFU/g, but after the in vitro test, the viable cell loss was too great to provide probiotic benefits. However, additional analysis of 10^9^ CFU/g of *Bifidobacterium animalis* subsp. *lactis* BB-12 added to yogurt ice cream revealed a viable cell count < 1 log CFU/g after the intestinal phase. Kawalczyk et al. (2022) reported a survival rate of 50.82% for *Bifidobacterium animalis* subsp. *Lactis* BB-12 after simulating in vitro digestion by adding 10^9^ CFU/g of *Bifidobacterium animalis* subsp. *Lactis* BB-12 to ice cream mixtures [22]. LGG in yogurt ice cream exhibited higher viability than did the other two strains for in vitro digestion.

Factors affecting probiotic survival in ice cream included probiotic strain, osmotic pressure, freeze injury, pH, overrun, oxygen scavengers, packaging, and frozen storage [23]. In this study, we selected *Lactobacillus acidophilus*, *Lacticaseibacillus rhamnosus*, and *Bifidobacterium animalis* subsp. *lactis,* which were the most common species of LAB used in probiotic ice cream for fermented dairy products. Effects of the overrun process can lead to decreased viable cell counts of probiotic bacteria. A previous study found that inclusion of 106–108% overrun decreased the viability of probiotics (*Lactobacillus acidophilus* LA5 and *Bifidobacterium animalis* subsp. *lactis* BB12) but by not more than 10% after freezing and hardening [24]. In addition, pH of the yogurt ice cream can affect the survival of tested probiotic strains. Acid stress can influence components of the probiotic cell membrane such as lipids and proteins, as well as damage the DNA and the chemical structure of peptidoglycan. These cellular level changes can inhibit bacterial proliferation. In this study, we did not measure overrun and pH values for each ice cream sample due to the focus on in vitro digestion, however these influences on probiotic viability are study limitations that could be further investigated.

### 3.3. Inhibition of α-Amylase and α-Glucosidase Activities by Probiotic Yogurt Ice Cream

**Figure 1:**
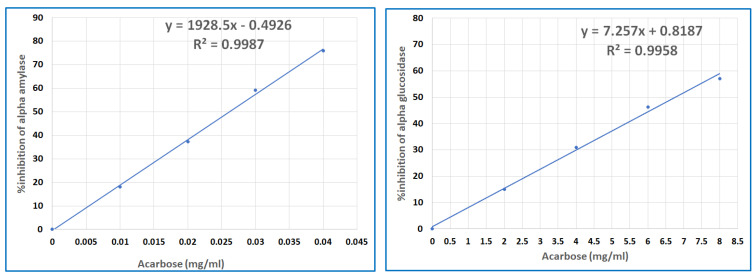
Inhibitory activities of acarbose on α-amylase and α-glucosidase.

Inhibiting α-amylase and α-glucosidase is the main mechanism of controlling postprandial hyperglycemia. We evaluated the inhibition of α-amylase and α-glucosidase via probiotic yogurt ice cream. Acarbose was used as a positive control because it functions as a carbohydrate digestion enzyme inhibitor by competing for binding sites and diminishing the enzymes’ affinity for oligosaccharides from dietary starch along with the monosaccharide production rate [25]. Figure 1 shows the data for the α-amylase and α-glucosidase inhibitory activities of acarbose. The 50% inhibitory concentration (IC50) of acarbose inhibited α-amylase and α-glucosidase at 0.026 mg/mL and 7.21 mg/mL, respectively. The results for α-glucosidase inhibitory activity by acarbose were consistent with those reported by Wihansah et al. (2018) with an IC50 value of 2.69 mg/mL in probiotic goat-milk yogurt supplemented with roselle extract [26].

The probiotics in the YIEs significantly differed in their ability to inhibit both enzymes (Table 2). The inhibition percentages for α-glucosidase activity from highest to lowest were YI-LA-5: 16.37% ± 0.32%, YI-LGG: 14.41% ± 0.26%, and YI-BB-12: 11.54% ± 0.21%. The inhibition percentages for α-amylase activity were YI-LGG: 41.37% ± 0.61%, YI-LA-5: 19.31% ± 0.30% and YI-BB-12: 16.14% ± 0.70%. An indirect measure of the antidiabetic potential of dairy products is the inhibition of α-amylase and α-glucosidase activities. The bioactive peptides resulting from the proteolytic enzymes produced by a particular probiotic strain can be attributed to an overall inhibition of both α-amylase and α-glucosidase [27].

Several factors may be responsible for α-amylase and α-glucosidase activities, including pH, different substrate, temperature, and the presence of any inhibitors or activators [28], and also quantities of peptides which interfere with α-amylase and α-glucosidase catalytic functions [29]. However, we did not measure peptides in our samples. Further research could identify potential bioactive peptides with inhibitory actions against α-amylase and α-glucosidase.

Lactic acid bacteria (LAB) can ferment sugar into lactic acid and produce proteolytic enzymes. By supplying nitrogen molecules, these enzymes play important roles in LAB growth. Protein in yogurt ice cream may also play a role in LAB growth and influence the inhibitory effects on α-amylase and α-glucosidase activities. Variations in the α-amylase and α-glucosidase inhibition percentages can be explained by the total protein contents in different types of milk. LAB possess a complex system of proteinases and peptidases that allow them to break down milk casein into small peptides. Peptides possess inhibitory potential against α-glucosidase and α-amylase, and their probable mechanism of action involves forming hydrophobic bonds at the enzyme’s active site [10,30]. In addition, a previous study evaluated potential antidiabetic strains of 14 candidate *Lactobacillus* subsp. strains through dipeptidyl peptidase IV (DPP-IV) inhibitory and antioxidant activities. The results of the experiment demonstrated the significant DPP-IV inhibitory activity and probiotic qualities of *L*. *acidophilus* KLDS1.0901 in vitro. In T2D mice, administration of *L*. *acidophilus* KLDS1.0901 could improve oxidative stress and insulin resistance while preserving blood glucose homeostasis [31].

### 3.4. Antioxidant Activity of Probiotics in Yogurt Ice Cream

Some probiotic strains possess antioxidative activity and reduce free radical accumulation by modulating the redox status of the host via an antioxidant system [13]. We measured the antioxidant activity of the YIEs using the DPPH and FRAP assays, which are simple, rapid, and convenient methods. One gram of yogurt ice cream with LA5, BB12, and LGG exhibited antioxidant ability using the DPPH assay equivalent to 21.40 ± 0.37, 23.95 ± 0.28, and 23.50 ± 0.33 mg of Trolox, respectively. For FRAP assay, 1 g of yogurt ice cream with LA5, BB12, and LGG demonstrated antioxidant ability equivalent to 97.63 ± 0.25, 67.31 ± 0.17, and 54.55 ± 0.4 mg of Trolox, respectively. The results of these assays showed that all YIEs with probiotics had significantly higher DPPH and FRAP values than the YIE-CT control, except for the DPPH value of YIE-LA-5, which did not differ from that of the control (Table 3). The differences in the antioxidant activities with different probiotics may be caused by differences in the strains ability to scavenge hydroxyl radicals and superoxide anions and produce antioxidants, as observed in our results. For the DPPH assay, YIE- BB12 and YIE-LGG showed significantly more ability to function as free-radical scavengers and hydrogen suppliers compared to YIE-LA-5. In the present study, LA5 exhibited the highest antioxidant activity (or reductants) in a sample that represented the corresponding concentration of electron-donating antioxidants with the reduction in the ferric iron (Fe3+) to the ferrous ion (Fe2+), compared to the other two probiotics.

Our finding is similar to that of Zahrani et al. (2023), who observed that all the fermented soy- and almond-based milks that they prepared by inoculation with single probiotic strains (*Lacticaseibacillus rhamnosus*, *Lactobacillus acidophilus, Lactiplantibacillus plantarum* and *Lacticaseibacillus casei*) had higher percentages of scavenging activity than did the unfermented control [32]. Furthermore, Abubakr et al. (2012) found that whey skim milk fermented with different LAB strain isolates with good proteolytic activity had better antioxidant activity than did the control skim milk, as determined by the DPPH assay [33]. YIE-LA-5 had a higher FRAP value than did YIE-LGG and YIE-BB-12, which is consistent with the findings of Ozcan et al. (2017), who reported that fermented milk supplemented with chestnut flour and *Lactobacillus acidophilus* had a higher FRAP value than did fermented milk supplemented with *Bifidobacterium animalis subsp*. *lactis* and *Lacticaseibacillus rhamnosus* [13]. Several mechanisms are responsible for antioxidant properties in probiotics, including scavenging hydroxyl radicals and superoxide anions, producing antioxidants and preventing lipid peroxidation [34]. Najgebauer-Lejko et al. (2014) reported the antioxidant property of *Lactobacillus acidophilus* LA-5 with FRAP and DPPH methods in a dose-dependent manner [35].

Raw milk antioxidant activity is meaningful for consumer health and can be enhanced by the antioxidant potential of bioactive peptides from probiotics and antioxidant properties of the probiotics themselves [36]. Casein, which is rich in potentially antioxidative amino acids such as tyrosine, tryptophan, histidine, lysine, and methionine, has been shown to scavenge superoxide anions, DPPH, and hydroxyl radicals and inhibit enzymatic and non-enzymatic lipid peroxidation, most likely through a free radical-scavenging mechanism [37]. One crucial factor influencing the antioxidant properties in fermented dairy products is the variations in protein content and the probiotic bacterial strain used. The total protein content in a dairy product plays a critical role in supporting LAB growth. LAB can ferment sugar into lactic acid and produce proteolytic enzymes, which use milk casein as a source of amino acids and peptides [30]. Peptides possess inhibitory potential against α-glucosidase and α-amylase, and their probable mechanism of action involves engaging in hydrophobic bonds at the enzyme’s active site and enhancing the antioxidant potential [38].

In vitro digestive systems are widely used to investigate and analyze changes, interactions, and the bioaccessibility of nutrients and non-nutritive compounds. This model is multipurpose, adaptable, accurate, and reproducible [39] and represents the physiological processes in the human digestive system. Of the three probiotics added to yogurt ice cream in this study, LGG showed the greatest benefit. However, the exact reasons for the decreased viability of the probiotic cells in the in vitro digestion system remain unclear owing to the specific enzymes and pH changes in the model.

## 4. Conclusions

The present study indicated that in the small intestine phase, YIE with *Lacticaseibacillus rhamnosus* had a higher probiotic content than 6 log CFU/g and therefore contributed evidence to support the concept that yogurt ice cream could provide a suitable matrix for the delivery of probiotics from dairy culture to enhance intestinal homeostasis with potential to promote probiotic benefits in the host. For antidiabetic properties, *Lactobacillus acidophilus* and *Lacticaseibacillus rhamnosus* showed higher levels of inhibition of α-glucosidase and α-amylase activities than *Bifidobacterium animalis* subsp. *lactis*. The beneficial effects of the three probiotics in yogurt ice cream as determined by DPPH and FRAP assays were higher than that of the control yogurt ice cream. These findings show that probiotic yogurt ice cream should be investigated in further studies as a potential functional food to help reduce post-prandial hyperglycaemia. Furthermore, the limitations of this investigation concerning overrun and pH effects, which impact the viability of probiotics in yogurt ice cream, should be assessed in conjunction with a comparative analysis of the inhibition of α-amylase and α-glucosidase by acarbose.

## Figures and Tables

**Table 1:** Viability of probiotics in yogurt ice cream before and during in vitro digestion.

Yogurt Ice Cream	Viable Probiotic Bacterial Counts in Yogurt Ice Cream (log CFU/g of Ice Cream)			
	Simulated In Vitro Digestive Stage			
	Before Digestion	Oral Phase	Gastric Phase	Intestinal Phase
YI-Control	ND	ND	ND	ND
YI-LA-5	6.61 ± 0.02 ^Ab^	5.12 ± 0.02 ^Bb^	4.65 ± 0.09 ^Cb^	4.11 ± 0.04 ^Db^
YI-BB-12	6.23 ± 0.03 ^Ac^	4.53 ± 0.57 ^Bb^	3.11 ± 0.12 ^Cc^	ND
YI-LGG	7.68 ± 0.04 ^Aa^	6.35 ± 0.09 ^Ba^	6.23 ± 0.08 ^Ba^	6.04 ± 0.03 ^Ca^

YI; yogurt ice cream, ND denotes not detected. ^a, b, c^ Different superscript lowercase letters denote significant differences (*p* < 0.05) between groups. ^A, B, C^ Different superscript uppercase letters denote significant differences (*p* < 0.05) within groups.

**Table 2:** Inhibition percentages of α-amylase and α-glucosidase by probiotic yogurt ice cream water extract (YIE) compared with the YIE control.

Yogurt Ice Cream	α-Amylase Inhibition (%)	α-Glucosidase Inhibition (%)
YIE-LA-5	19.31 ± 0.30 ^b^	16.37 ± 0.32 ^a^
YIE-BB-12	16.14 ± 0.70 ^c^	11.54 ± 0.21 ^c^
YIE-LGG	41.37 ± 0.61 ^a^	14.41 ± 0.26 ^b^

YIE; yogurt ice cream water extract. ^a, b, c^ Different superscript lowercase letters denote significant differences (*p* < 0.05) between groups.

**Table 3:** Antioxidant properties of water extract probiotic yogurt ice cream measured by DPPH-radical scavenging and the FRAP method.

Yogurt Ice Cream	DPPH (mg Trolox Equivalent/g of Sample)	FRAP (mg Trolox Equivalent/g of Sample)
YIE-CT	21.40 ± 0.40 ^b^	51.47 ± 0.23 ^d^
YIE-LA-5	21.40 ± 0.37 ^b^	97.63 ± 0.25 ^a^
YIE-BB-12	23.95 ± 0.28 ^a^	67.31 ± 0.17 ^b^
YIE-LGG	23.50 ± 0.33 ^a^	54.55 ± 0.40 ^c^

YIE; yogurt ice cream water extract. ^a, b, c, d^ Different superscript lowercase letters denote significant differences (*p* < 0.05) between groups.

## Data Availability

The data presented in this study are available on request from the corresponding author.
